# Growth Retardation in an Adolescent Secondary to Cushing’s Disease Caused by a Pituitary Microadenoma: A Case Report

**DOI:** 10.3390/reports9010070

**Published:** 2026-02-26

**Authors:** María Cristina Ontoria Betancort, Inés García de Pablo, Cristina Díaz Martín, Sebastián Eustaquio Martín Pérez, Isidro Miguel Martín Pérez

**Affiliations:** 1 Servicio de Pediatría, Hospital Universitario Nuestra Señora de Candelaria, 38010 Santa Cruz de Tenerife, Spain; 2 Faculty of Health Sciences, Universidad Europea de Canarias, 38300 La Orotava, Santa Cruz de Tenerife, Spain; 3 Faculty of Medicine, Health and Sports, Universidad Europea de Madrid, 28670 Villaviciosa de Odón, Madrid, Spain; 4 Faculty of Health Sciences, Universidad del Atlántico Medio, 35017 Tafira Baja, Las Palmas, Spain

**Keywords:** Cushing’s disease, growth retardation, pituitary microadenoma, pediatric endocrinology

## Abstract

**Introduction and Clinical Significance**: Cushing’s disease is a rare but clinically consequential cause of endogenous hypercortisolism in children, most commonly resulting from ACTH-secreting pituitary microadenomas. In contrast to adults, growth deceleration accompanied by disproportionate weight gain represents the earliest and most sensitive clinical marker in pediatric cases. Sustained hypercortisolism during critical periods of somatic maturation may compromise final height, disrupt pubertal progression, and induce persistent metabolic and neurocognitive sequelae, even after biochemical remission. Early recognition and timely intervention are, therefore, essential to preserve developmental trajectories. **Case Presentation**: A 13-year-and-8-month-old boy was referred for evaluation of progressive linear growth impairment, markedly reduced growth velocity (0.8 cm/year; <1st percentile), and insidious weight gain over a two-year period. His height was at the 5th percentile, substantially below the mid-parental target. Biochemical assessment showed repeated elevations of 24 h urinary free cortisol and ACTH levels, consistent with ACTH-dependent hypercortisolism. Dynamic testing supported a pituitary etiology, and high-resolution MRI identified a 3 × 2 mm microadenoma. The patient underwent successful endoscopic transsphenoidal resection. Postoperatively, transient central diabetes insipidus and secondary adrenal insufficiency developed, requiring structured endocrine follow-up. Recovery of hypothalamic–pituitary–adrenal axis function was confirmed one year after surgery, allowing discontinuation of glucocorticoid replacement. **Conclusions**: Cushing’s disease should be suspected in children presenting with growth deceleration in the context of disproportionate weight gain. Timely diagnosis and early surgical management are essential to mitigate long-term auxological and metabolic sequelae. Postoperative endocrine disturbances, particularly transient adrenal insufficiency, are frequent and require systematic follow-up with periodic functional reassessment to ensure complete endocrine recovery.

## 1. Introduction and Clinical Significance

Cushing’s disease constitutes a rare yet profoundly consequential cause of endogenous hypercortisolism in the pediatric population, most commonly arising from an adrenocorticotropic hormone (ACTH)-secreting pituitary adenoma [1,2]. Despite its low incidence in childhood, Cushing’s disease poses a major diagnostic and therapeutic challenge due to its insidious onset and the frequently nonspecific nature of early symptoms [3].

In contrast to adults—among whom central adiposity and metabolic derangements typically dominate the clinical picture—growth failure emerges as the most sensitive and clinically informative early marker of Cushing’s disease in children [4]. The paradoxical coexistence of progressive weight gain with declining linear growth reflects the profound catabolic effects of cortisol excess on the developing skeleton and should immediately prompt endocrine evaluation [5,6]. Importantly, sustained hypercortisolism during critical periods of somatic maturation may result in long-term biological consequences. These long-term consequences may include impaired final height attainment, disruption of normal pubertal development, adverse cardiometabolic reprogramming, and persistent neurocognitive vulnerability that may endure even after biochemical remission [7,8,9].

From a developmental perspective, the clinical gravity of pediatric Cushing’s disease lies in the susceptibility of the developing organism to chronic glucocorticoid exposure [10,11]. Excess cortisol exerts multisystem toxicity, hypertension, insulin resistance, dyslipidemia, impaired bone growth, delayed sexual maturation, and a spectrum of neuropsychiatric disturbances [12,13]. When diagnosis is delayed, these disturbances may extend beyond childhood, predisposing affected individuals to premature cardiovascular disease, skeletal fragility, and reduced health-related quality of life [14,15,16,17]. Accordingly, early recognition and timely therapeutic intervention are not only desirable but biologically imperative to preserve normal developmental trajectories.

Pathophysiologically, Cushing’s disease arises as a consequence of autonomous ACTH hypersecretion, typically driven by a corticotroph pituitary adenoma, resulting in sustained adrenal stimulation, bilateral adrenal hyperplasia, and chronic cortisol overproduction [14]. Persistent glucocorticoid excess exerts pleiotropic systemic effects, altering carbohydrate, protein, and lipid metabolism, impairing immune homeostasis, and disrupting the physiological feedback of the hypothalamic–pituitary–adrenal (HPA) axis [18]. In the pediatric setting, prolonged hypercortisolism interferes with the tightly regulated processes of somatic growth and pubertal maturation, thereby amplifying its developmental impact during critical windows of biological vulnerability.

From a diagnostic standpoint, the evaluation of pediatric Cushing’s disease integrates careful clinical suspicion with biochemical confirmation of hypercortisolism and targeted neuroimaging. Standard laboratory evaluation includes 24 h urinary free cortisol (UFC), late-night salivary or serum cortisol levels, and low-dose dexamethasone suppression testing (DST) to establish endogenous cortisol excess [19]. Subsequent evaluation with ACTH measurement and high-resolution pituitary magnetic resonance imaging (MRI) is essential for localizing the source of hormone secretion [20]. Nevertheless, the diminutive size of corticotroph microadenomas in children often limits imaging sensitivity, occasionally generating diagnostic uncertainty and necessitating repeated or advanced imaging strategies [4].

Therapeutically, endoscopic endonasal transsphenoidal surgery (EETS) remains the cornerstone of treatment for pediatric Cushing’s disease and is associated with high remission rates when performed in specialized, high-volume centers [15,21]. However, surgical cure often comes at the cost of transient or prolonged endocrine disequilibrium [22]. Secondary adrenal insufficiency and central diabetes insipidus (CDI) are among the most frequently postoperative disturbances, largely reflecting the consequence of prior suppression and subsequent recovery of the HPA axis [12,14,18]. These sequelae may be particularly relevant in children and adolescents, whose endocrine systems are inherently dynamic and vulnerable. Consequently, meticulous long-term endocrine surveillance is indispensable to ensure timely identification of hormonal deficits and to support optimal somatic recovery [13].

Although pediatric Cushing’s disease secondary to pituitary microadenomas has been extensively documented, several aspects of its developmental trajectory remain insufficiently characterized [23,24]. Within this context, the present case is distinguished by three clinically relevant features. First, the patient exhibited uncommonly severe and prolonged growth retardation prior to diagnosis, suggesting sustained exposure to hypercortisolism during a critical developmental period. Second, the onset of cortisol excess occurred at a pivotal stage of pubertal maturation, potentially compromising normal height progression and final stature attainment. Third, the clinical course was characterized by a prolonged period of postoperative endocrine dysfunction before complete recovery of the HPA axis was achieved.

Beyond illustrating diagnostic challenges and surgical management of pediatric Cushing’s disease, this report contributes a longitudinal perspective on endocrine recovery and somatic outcomes following treatment. By emphasizing the interplay between diagnostic timing, endocrine disruption, and growth dynamics, this case reinforces the critical importance of early recognition, accurate diagnosis, multidisciplinary management, and structured follow-up to optimize developmental outcomes in such patients.

## 2. Case Presentation

### 2.1. Patient Information

A 13-year-and-8-month-old male was referred for specialized endocrine evaluation due to progressive linear growth impairment, a pronounced decline in growth velocity, and insidious weight gain evolving over the preceding 12–24 months. At presentation, his height corresponded to the 5th percentile for age, substantially below the mid-parental target height of 177.5 cm—the 51st percentile—thereby raising concern for pathological growth attenuation.

With respect to perinatal and developmental history, the patient was born at term with anthropometric parameters appropriate for gestational age and achieved all neurodevelopmental milestones within expected chronological ranges. Pubertal onset occurred spontaneously at 11 years of age, consistent with physiological timing. The child’s past medical and surgical history was unremarkable, and no family history of endocrine, metabolic, or hereditary disorders was reported. Furthermore, no prior exposures, chronic illnesses, or pharmacological treatments known to interfere with HPA axis function were identified.

From a neurocognitive standpoint, the patient showed high academic achievement without evidence of learning or attentional deficits. However, according to caregiver reports, a progressive decline in school performance emerged over the two years surrounding diagnosis and surgical management, primarily characterized by impaired sustained attention and reduced concentration capacity. Notably, these difficulties persisted throughout the active phase of hypercortisolism and extended into the early postoperative period. Demographic and clinical characteristics are summarized in Table 1.

### 2.2. Clinical Findings

On physical examination, multiple phenotypic features suggestive of endogenous hypercortisolism were observed. The patient exhibited marked central adiposity with disproportionate truncal fat distribution, adipomastia, rounded facies accompanied by facial plethora, and a prominent dorsocervical fat pad (“buffalo hump”). In addition, dermatological assessment revealed inflammatory and nodulocystic acne affecting the facial and upper thoracic regions, along with fine hypertrichosis over the upper back. Notably, classical stigmata such as violaceous striae, proximal myopathy, or spontaneous ecchymoses were absent. Blood pressure values remained within age- and sex-specific reference ranges, and neurological examination demonstrated no focal deficits.

From an auxological perspective, body weight was 52.4 kg (41st percentile; SDS −0.24), height was 148.6 cm (5th percentile; SDS −1.68), and BMI was 23.7 kg/m^2^ (79th percentile; SDS +0.82). Linear growth velocity was profoundly impaired at 0.8 cm/year (<1st percentile; SDS −8.07), consistent with severe growth suppression. Pubertal staging corresponded to Tanner stage III, and bilateral testicular volumes measured 15 mL. Skeletal maturation, assessed according to the Greulich and Pyle atlas (G&P Atlas), revealed a delayed bone age (BA) of 11.5 years compared with a chronological age (CA) of 13 years and 2 months. Predicted adult height, calculated using the Bayley–Pinneau (B&P) method, was 178.6 cm, consistent with genetic target height expectations.

With regard to laboratory findings, hematological and biochemical tests were also within normal limits. Thyroid function was preserved, as serum thyroid-stimulating hormone (TSH) and free thyroxine (FT4) levels were within reference ranges. Likewise, the GH–IGF axis was unaltered, with age- and pubertal stage-appropriate insulin-like growth factor 1 (IGF-1) and insulin-like growth factor binding protein 3 (IGFBP-3) concentrations.

Gonadotropin and total testosterone concentrations were consistent with pubertal maturation. In contrast, evaluation of the adrenal axis demonstrated markedly elevated 24 h urinary free cortisol (UFC) levels across three independent collections (348.8 µg/24 h, 1255.3 µg/24 h, and 575.64 µg/24 h), corresponding to three-to-five-fold elevations above the upper reference limit. Basal morning serum cortisol measured 16.8 µg/dL, and plasma ACTH was detectable at 21.9 pg/mL Complementary results are summarized in Table 2.

### 2.3. Timeline

Physiological pubertal onset occurred at 11 years of age, representing the last documented phase of normal growth and developmental progression. Subsequently, progressive weight gain and attenuation of linear growth became clinically apparent. Over the ensuing 12–24 months, growth failure progressively intensified, while characteristic Cushingoid features gradually emerged. Concomitantly, a decline in academic performance and sustained attention was observed, paralleling the evolution of hypercortisolism.

At 13.7 years of age, a comprehensive endocrine evaluation confirmed endogenous hypercortisolism. Subsequent sellar MRI identified a pituitary microadenoma consistent with Cushing’s disease, and the patient underwent EETS. During the first postoperative year, marked clinical improvement was observed, including normalization of growth velocity and regression of hypercortisolism-related phenotypic features. Ultimately, final adult height was achieved at 18 years of age, prior to transition to adult endocrinology care. The chronological clinical course is summarized in Table 3.

In this clinical context, the high-dose (8 mg) DST was pivotal in the differential diagnosis. Cortisol decreased from 16.9 µg/dL to 1.12 µg/dL (>90% suppression), a pattern characteristic of pituitary corticotroph adenoma and atypical of adrenal cortisol-producing tumors, which generally fail to suppress. The absence of biochemical evidence suggestive of adrenal autonomy, together with the sustained postoperative remission and documented recovery of the HPA axis following tumor resection, ultimately confirmed the etiological diagnosis of Cushing’s disease.

**Figure 1:**
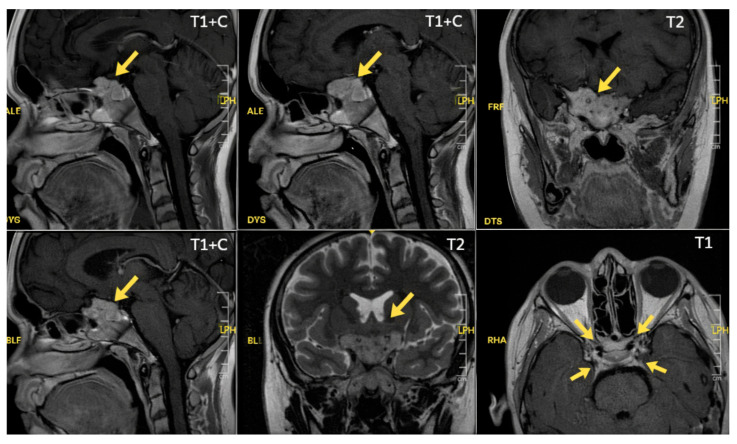
Magnetic resonance imaging (MRI) of the sellar region in a pediatric patient with Cushing syndrome shows a small nodular lesion along the left margin of the sella turcica (approximately 3 × 2 mm). The lesion appears hyperintense on T2-weighted sequences, isointense on pre-contrast T1-weighted images, and exhibits progressive enhancement following contrast administration, imaging features consistent with a pituitary microadenoma (arrows). The posterior pituitary bright spot, pituitary stalk (infundibulum), and hypothalamic structures show normal morphology and signal intensity. Source: Own work.

Furthermore, high-resolution pituitary MRI was performed using dedicated sellar protocols, including thin-slice T1-weighted sequences before and after gadolinium administration, as well as dynamic contrast-enhanced imaging. These studies identified a focal hypoenhancing lesion measuring 3 × 2 mm along the left lateral margin of the sella turcica, consistent with a pituitary microadenoma. Additional imaging findings are presented in Figure 1.

The patient underwent EETS, which was fully completed without intraoperative complications. No cerebrospinal fluid leakage, vascular injury, or damage to adjacent neurovascular structures was observed. Gross total resection was achieved, and histopathological analysis confirmed a corticotroph pituitary neuroendocrine tumor (PitNET) with immunohistochemical positivity for ACTH, definitively establishing the pituitary origin of cortisol excess.

Perioperative glucocorticoid coverage was administered in accordance with a standardized stress-dose protocol to prevent adrenal crisis. Intravenous hydrocortisone (100 mg) was administered at induction of anesthesia, followed by 50 mg every 6 h during the first 24 postoperative hours. The regimen was progressively tapered as the patient remained hemodynamically stable and resumed oral intake. Thereafter, physiological oral hydrocortisone replacement was initiated at 10 mg/m^2^/day and maintained until recovery of HPA axis function was confirmed by a normal response to the standard ACTH (Synacthen^®^) stimulation test.

### 2.4. Follow-Up and Outcomes

At 10 months postoperatively, the patient exhibited marked clinical and metabolic improvement. BMI normalized, and significant catch-up growth was observed, with a height gain of 12 cm and restoration of age-appropriate growth velocity (>6 cm/year). Cushingoid features—including moon facies, truncal obesity, dorsocervical fat pad, and inflammatory acne—resolved completely. Likewise, caregiver reports also described a progressive improvement in attention, academic performance, and overall psychosocial functioning following disease remission.

At 18 years of age, the patient reached a final adult height of 167.5 cm (SDS −1.22; 12th percentile). Despite remaining within the normal range, final height was below both the mid-parental target height and the initially predicted adult height, suggesting a partial and likely irreversible impact of prolonged hypercortisolism on linear growth despite adequate biochemical remission. At transition to adult endocrinology care, the patient had achieved complete pubertal maturation (Tanner stage V), showed normal HPA axis function on dynamic testing, which did not require ongoing glucocorticoid replacement therapy.

## 3. Discussion

Cushing’s disease is an uncommon yet clinically significant cause of endogenous hypercortisolism, often posing a diagnostic challenge due to its insidious onset and progressive course in children [19]. Unlike adults, in whom metabolic complications frequently predominate, growth failure represents the most sensitive and clinically informative early manifestation of Cushing’s disease in children and adolescents. The present case exemplifies this hallmark pattern, characterized by a marked dissociation between progressive weight gain and profound impairment of linear growth.

According to prior evidence, multiple pediatric cohorts have shown that a reduction in growth velocity below the 3rd percentile—particularly when accompanied by weight gain—should promptly raise suspicion for hypercortisolism [21,22]. In our patient, growth velocity declined to 0.8 cm/year (<1st percentile), a degree of impairment rarely attributable to constitutional delay or exogenous obesity alone. Similar studies reported growth deceleration in more than 90% of affected children at diagnosis, often preceding overt Cushingoid features and serving as one of the earliest clinical indicators of disease [4,6]. Likewise, other studies have emphasized that impaired linear growth remains frequently underrecognized and represents a major contributor to diagnostic delay in pediatric Cushing’s disease [25].

From a pathophysiological standpoint, the epiphyseal plate represents a primary biological target of chronic hypercortisolism. Sustained glucocorticoid excess suppresses chondrocyte proliferation, impairs extracellular matrix synthesis, and accelerates senescence within the hypertrophic zone, disrupting endochondral ossification and longitudinal bone growth [26,27]. At both central and peripheral levels, cortisol inhibits the GH–IGF-1 axis by reducing GH pulsatility, attenuating hepatic IGF-1 production, and impairing IGF-1 bioactivity within the growth plate microenvironment [28,29].

During puberty, these inhibitory effects may be further amplified through interference with sex steroid-dependent regulation of growth, altering the physiological balance between proliferative expansion and estrogen-mediated epiphyseal maturation [30]. Prolonged exposure to hypercortisolism during critical developmental windows may induce structural and functional alterations of the growth plate that are only partially reversible, thereby limiting complete catch-up growth even after biochemical remission is achieved [31]. This mechanism provides a plausible explanation for the persistent growth impairment observed in some patients despite successful treatment of pediatric Cushing’s disease, as prior prolonged exposure to hypercortisolism may result in lasting alterations in normal growth processes.

From a diagnostic perspective, the diagnostic evaluation of this patient was consistent with contemporary clinical recommendations. Repeated elevation of UFC confirmed endogenous hypercortisolism, while the absence of cortisol suppression following DST supported an ACTH-dependent etiology [32,33]. Importantly, merging evidence suggests that mild hypercortisolism—potentially more prevalent in pediatric populations than previously recognized—may complicate biochemical interpretation and therefore necessitate repeated testing within an appropriate clinical context [24].

High-resolution pituitary MRI revealed a 3 × 2 mm hypoenhancing lesion consistent with a corticotroph microadenoma, a size characteristic of pediatric Cushing’s disease. However, this conventional medical image demonstrates limited sensitivity for detecting ACTH-secreting microadenomas in children, and up to 40% of cases may lack a clear lesion [22,25,33]. Advances in imaging, including dynamic contrast-enhanced sequences and high-field MRI, have improved detection rates through enhanced spatial resolution and lesion conspicuity. Diagnostic decision-making should therefore rely on the overall concordance of clinical, biochemical, and radiological findings, which together form the cornerstone of etiological assessment [20].

In this context, inferior petrosal sinus sampling (IPSS) is widely regarded as the reference standard for confirming the central origin of ACTH secretion, particularly in cases with inconclusive imaging [34]. However, it was not pursued in the present case because the biochemical profile was consistent with ACTH-dependent hypercortisolism, and high-resolution MRI showed a discrete focal lesion compatible with a corticotroph microadenoma [35,36]. Accordingly, when biochemical and radiological findings are concordant, current evidence supports a selective rather than systematic application of IPSS.

Furthermore, given the invasive nature of the procedure and the potential risks associated with venous catheterization in pediatric patients—including vascular injury and thrombotic complications—the multidisciplinary team determined that IPSS was unlikely to provide additional clinically actionable information [37,38]. Therefore, the diagnostic strategy was individualized through a structured risk–benefit assessment, prioritizing patient safety without compromising diagnostic certainty. Importantly, sustained postoperative remission further validated the appropriateness of this tailored approach.

Therapeutically, endoscopic endonasal transsphenoidal surgery (EETS) represents the first-line treatment for pediatric Cushing’s disease and achieves remission rates ranging from 65% to 90% when performed in experienced, high-volume centers [33,39]. The postoperative course observed reflects well-recognized endocrine sequelae of curative surgery. For instance, transient CDI is a known complication of pituitary procedures in children and typically resolves within months [16,33,40]. Similarly, postoperative adrenal insufficiency primarily reflects chronic suppression of the HPA axis and is often regarded as a favorable biochemical marker of surgical remission [41].

Notably, the patient showed substantial catch-up growth during follow-up, gaining 12 cm over 10 months with normalization of growth velocity. Early diagnosis and timely surgical intervention are consistently identified as key determinants of growth recovery, whereas prolonged exposure to cortisol excess may irreversibly compromise final adult height [6,41]. Although adjunctive growth hormone (GH) therapy may be warranted in selected patients, the spontaneous growth recovery—as observed here—underscores effective disease control as the principal driver of somatic restoration [42].

Nevertheless, despite this encouraging acceleration, the patient ultimately failed to reach his mid-parental target height. This outcome reinforces the concept of a “window of irreversible damage,” during which sustained hypercortisolism—particularly during puberty—may induce lasting structural and functional impairment of the growth plate [3,21]. In this regard, the estimated symptom duration of 12 to 24 months in this case likely overlapped with a particularly vulnerable maturational phase, thereby limiting the potential for complete growth recovery despite biochemical remission.

Beyond auxological considerations, this case underscores the neurocognitive burden associated with pediatric Cushing’s disease [43]. Although academic performance had been strong prior to disease onset, the patient developed measurable impairments in sustained attention and academic functioning during the peri-diagnostic period. Accumulating evidence indicates that chronic hypercortisolism during childhood may induce structural and functional alterations in fronto-limbic circuitry, hippocampal integrity, and cortical maturation, with potential long-term consequences for executive function and cognitive processing [6,44,45]. While inherently limited by its single-case design, this report nonetheless provides clinically meaningful insight into the developmental repercussions of delayed recognition.

In summary, this case report highlights the importance of maintaining a high index of clinical suspicion for Cushing’s disease in children presenting with impaired linear growth accompanied by disproportionate weight gain. By providing a longitudinal view of disease progression, treatment, and endocrine recovery, the report contributes clinically relevant evidence regarding the impact of diagnostic timing on developmental outcomes. These findings support the adoption of comprehensive diagnostic strategies, individualized decision-making, and early surgical management to minimize long-term consequences. Future studies should aim to establish more precise pediatric diagnostic criteria, improve the sensitivity of imaging approaches, and identify factors associated with long-term somatic, neurocognitive, and psychosocial recovery in affected patients.

### Patient Perspective

The patient described the period preceding diagnosis as one marked by progressive physical changes and declining academic performance, both of which negatively affected his self-esteem and psychosocial well-being. The gradual alteration in body habitus and reduced sustained attention were perceived as particularly distressing. Although the prospect of surgical intervention generated understandable anxiety, he reported feeling adequately informed and supported by the multidisciplinary team throughout the diagnostic and therapeutic process.

Following EETS, he experienced a substantial improvement in overall well-being. He noted increased energy levels, progressive normalization of physical appearance, and enhanced concentration and academic performance. While he expressed some disappointment that his final adult height did not fully reach the mid-parental target, he conveyed overall satisfaction with the treatment received and acknowledged its positive impact on his health, functional capacity, and quality of life.

## 4. Conclusions

Cushing’s disease, although rare in children, should be suspected in the presence of growth deceleration associated with disproportionate weight gain. Early recognition and prompt surgical management are essential to reduce the risk of irreversible effects on linear growth, pubertal development, and metabolic health. Postoperative secondary adrenal insufficiency is common but typically transient, highlighting the need for careful glucocorticoid replacement and structured long-term endocrine follow-up to ensure complete recovery and optimal outcomes.

## Figures and Tables

**Table 1:** Demographic and clinical characteristics.

Parameter	Results
Sex	Male
Age	13.7 years
Clinical presentation	Progressive growth failure, reduced growth velocity, and weight gain
Symptom duration	12–24 months
Height status	5th percentile (below mid-parental target)
Mid-parental height	177.5 cm (51st percentile)
Birth and development	Term birth, appropriate for gestational age, normal neurodevelopment
Pubertal onset	Physiological onset at 11 years
Medical history	No relevant personal or family history
Endocrine risk factors	No known factors affecting HPA axis

Complementary tests were grouped by physiological systems in a 13.7-year-old male with progressive growth failure, reduced growth velocity, and weight gain. Height was at the 5th percentile, below the mid-parental target (177.5 cm; 51st percentile). Birth, neurodevelopment, and pubertal onset were normal, with no relevant medical history or HPA axis risk factors.

**Table 2:** Complementary test results grouped by physiological systems.

System	Parameter	Result	Interpretation
Anthropometric	Weight	52.4 (SDS −0.24) kg, p41	Normal
	Height	148.6 (SDS −1.68) cm, p5	↓ Reduced for age
	BMI	23.73 (SDS +0.82) kg/m^2^, p75	Normal–high
	Growth velocity	0.8 (SDS −8.07) cm/year, *p* < 1	↓ Severely reduced
Skeletal maturation	Chronological age	13 years and 2 months	
	Bone age (G&P Atlas)	11 years and 6 months	↓ Delayed
	Predicted adult height (B&P method)	178.6 cm	Within expectation
Hematology	Hemoglobin	14.2 g/dL (13.0–17.0 g/dL)	Normal
	Hematocrit	42% (39–50%)	Normal
	RBC	4.9 × 10^6^/µL (4.5–5.9 × 10^6^/µL)	Normal
	WBC	6.8 × 10^3^/µL (4.0–10.0 × 10^3^/µL)	Normal
	Platelets	278 × 10^3^/µL (150–400 × 10^3^/µL)	Normal
Metabolic panel	Glucose	96 mg/dL (70–99 mg/dL)	Normal
	BUN	18 mg/dL (7–20 mg/dL)	Normal
	Creatinine	0.8 mg/dL (0.6–1.2 mg/dL)	Normal
	Sodium	141 mmol/L (135–145 mmol/L)	Normal
	Potassium	4.2 mmol/L (3.5–5.1 mmol/L)	Normal
	Chloride	103 mmol/L (98–107 mmol/L)	Normal
	Total calcium	9.4 mg/dL (8.6–10.2 mg/dL)	Normal
	GOT	28 U/L (<40 U/L)	Normal
	GPT	31 U/L (<41 U/L)	Normal
	Alkaline phosphatase	312 U/L (130–525 U/L)	Normal
	Total bilirubin	0.7 mg/dL (0.3–1.2 mg/dL)	Normal
	Total protein	7.2 g/dL (6.0–8.3 g/dL)	Normal
	Albumin	4.4 g/dL (3.5–5.0 g/dL)	Normal
Thyroid axis	TSH	2.1 mIU/L (0.5–4.5 mIU/L)	Normal
	FT4	1.2 ng/dL (0.8–1.8 ng/dL)	Normal
GH–IGF axis	IGF–1	491.10 ng/mL (202–957 ng/mL)	Normal
	IGFBP–3	2.19 µg/mL (1.73–5.11 µg/mL)	Normal
Gonadal axis	FSH	4.22 mIU/mL (5–10 mIU/mL)	Consistent with puberty
	LH	0.63 mIU/mL	Early pubertal range
	Total testosterone	1.83 ng/mL	Pubertal
Adrenal axis	Serum cortisol	16.8 µg/dL (5–25 µg/dL)	Normal (non-suppressed)
	ACTH	21.9 pg/mL (10–60 pg/mL)	Normal
	UFC 1 (*24 h*)	348.8 µg/24 h (235.6 µg/m^2^/24 h)	↑ Markedly elevated
	UFC 2 (*24 h*)	1255.3 µg/24 h (845.47 µg/m^2^/24 h)	↑↑ Markedly elevated
	UFC 3 (*24 h*)	575.64 µg/24 h (319 µg/m^2^/24 h)	↑ Markedly elevated
Dynamic Test	Low-dose DST (1 mg)	Basal: 16.8 µg/dL (5–18 µg/dL) Post: 1.86 µg/dL % Suppression: 88.9% (<1.8 µg/dL)	Borderline suppression
	High-dose DST (8 mg)	Basal: 16.9 µg/dL (5–18 µg/dL) Post: 1.12 µg/dL % Suppression: 93.4% (≥50% reduction)	Appropriate suppression (pituitary pattern)
Gastroenterology	Celiac disease screening	Negative	Normal

Clinical, anthropometric, skeletal, biochemical, and hormonal parameters at diagnosis are presented. Chronological age (CA) is expressed in decimal years; bone age (BA, Greulich and Pyle) and predicted adult height (PAH, Bayley–Pinneau) were assessed. Anthropometric data are reported as absolute values with SDS and percentiles. Urinary free cortisol (UFC) was obtained from three 24 h collections, and dynamic evaluation included 1 mg and 8 mg dexamethasone suppression tests (DST). Abbreviations: ACTH, adrenocorticotropic hormone; BA, bone age; BMI, body mass index; CA, chronological age; DST, dexamethasone suppression test; PAH, predicted adult height; SDS, standard deviation score; UFC, urinary free cortisol.

**Table 3:** Chronological clinical course from pubertal onset to final adult height.

Age	Event	Clinical Significance
11 yrs.	Pubertal onset	Early pubertal activation suspected
12–13 yrs.	Growth deceleration & weight gain	Possible endocrine dysfunction
13.7 yrs.	Endocrine evaluation & diagnosis	Hormonal assessment confirms pathology
13.8 yrs.	Transsphenoidal surgery	Surgical intervention for pituitary lesion
>10 months	Catch-up growth	Recovery of growth velocity post-treatment
18 yrs.	Final adult height	Definitive height outcome assessment

Chronological clinical course from pubertal onset to final adult height. The interval “+10 months” indicates the time elapsed after EETS. Abbreviation: yrs, years.

## Data Availability

The original contributions presented in this study are included in the article. Further inquiries can be directed to the corresponding author.

## Abbreviations

The abbreviations used throughout this manuscript are as follows:

ACTH	Adrenocorticotropic hormone
ALT (GPT)	Alanine aminotransferase
AST (GOT)	Aspartate aminotransferase
BA	Bone age
BMI	Body mass index
BP	Bayley–Pinneau
BUN	Blood urea nitrogen
CA	Chronological age
CDI	Central diabetes insipidus
DST	Dexamethasone suppression test
EETS	Endoscopic endonasal transsphenoidal surgery
FT4	Free thyroxine
FSH	Follicle-stimulating hormone
GH	Growth hormone
GP	Greulich and Pyle
IGF–1	Insulin-like growth factor 1
IGFBP–3	Insulin-like growth factor binding protein 3
IPSS	Inferior petrosal sinus sampling
LH	Luteinizing hormone
PAH	Predicted adult height
RBC	Red blood cells
TSH	Thyroid-stimulating hormone
UFC	Urinary free cortisol
WBCs	White blood cells
